# A Malformed Tooth

**Published:** 1872-11

**Authors:** 


*A Malformed Tooth.*—Dr. B. Smith of Churchville, Md., writes as follows: "I have a case of a lower, left lateral incisor, bent about one-third of its length, almost at a right angle, on its labial surface, to the annoyance of the patient, by the lip continually rubbing against it. By cutting it off, it will appear much shorter; and the advice of several dentists has been to let it alone; but it is too annoying and looks badly. If you think it worth a notice in the American Journal of Dental Science, I would be glad to have an opinion on it. By so doing you will very much oblige," &c.

[We should advise the removal of elongated part, which acts as an irritant to the lip, if this is possible without rendering the tooth sensitive; otherwise the extraction of the tooth is demanded.—Ed.]

